# No effect of nitrate-rich beetroot juice on microvascular function and blood pressure in younger and older individuals: a randomised, placebo-controlled double-blind pilot study

**DOI:** 10.1038/s41430-022-01115-4

**Published:** 2022-03-29

**Authors:** David Rogerson, Fabio Alejandro Aguilar Mora, Jamie Stuart Young, Markos Klonizakis

**Affiliations:** 1 Sport and Physical Activity Research Centre, Department of Sport and Physical Activity, Sheffield Hallam University, Sheffield, UK; 2 Lifestyle, Exercise and Nutrition Improvement (LENI) Research Group, Department of Nursing and Midwifery, Faculty of Health and Wellbeing, Sheffield Hallam University, Sheffield, UK; 3 The University of Sheffield, Department of Oncology & Metabolism, Sheffield, UK; 4 Biomolecular Sciences Research Centre, Sheffield Hallam University, Sheffield, UK

**Keywords:** Hypertension, Atherosclerosis

## Abstract

**Background/Objectives:**

To compare the effects of supplemental inorganic nitrate (NO_3_) on microvascular endothelial function and blood pressure in younger vs. older participants.

**Subjects/Methods:**

25 individuals participated in a double-blind, randomised, placebo-controlled crossover pilot study. Participants were stratified by age (18–35 and ≥55 years) and consumed a single dose beetroot juice (providing 6.4 mmol NO_3_) or NO_3_-depleted beetroot juice. Blood pressure, microvascular function (via Laser Doppler Flowmetry; LDF) and urinary NO_3_ were assessed, and the effects of NO_3_ supplementation on cardiovascular parameters were compared between participants and conditions using mixed-design ANOVA.

**Results:**

Treatments and methods were well tolerated, and no adverse events were reported. Urinary NO_3_ increased 3 h following ingestion in both groups, (*P* = 0.02). Levels remained elevated at 24 h post consumption in younger participants only (*P* = 0.02). Beetroot juice had no effect on blood pressure in either group nor on microcirculatory endothelial function.

**Conclusions:**

Beetroot juice had no effect on blood pressure or microvascular endothelial function in young and older individuals. Dosage and timing regimens for supplemental beetroot juice should be avenues for further inquiry.

## Introduction

Ageing is an important risk factor in cardiovascular disease, can lead to arterial stiffening, increased systolic and diastolic blood pressure (SBP and DBP) and the deterioration of the endothelium [1–3]. This is known as endothelial dysfunction, which is characterised by reduced vasodilation, increased inflammation, and thrombosis. Endothelial dysfunction is a precursor for chronic disease, typified by reduced nitric oxide (NO) supply, an imbalance in the contribution of endothelium-derived relaxing and contracting factors, and impaired angiogenesis [3, 4]. Endothelial dysfunction is treatable, particularly by modulating the supply of NO via dietary and pharmacological factors [5, 6].

NO is a paracrine signalling molecule that regulates blood vessel tone [6, 7]. NO is produced by enzymes called NO synthases, however NO can also be produced via the nitrate-nitrite-NO pathway [5–8]. Nitrate (NO_3_) was once considered an inert product of NO metabolism, however it is now understood to possess physiological properties [8, 9], being reduced enzymatically to produce NO [8, 9]. Thus, supplementation is considered as a promising new avenue in the pursuit of non-pharmacological cardiovascular treatments [10–14]. NO_3_ is found in vegetables, and beetroot appears to be a rich source. Beetroot juice is a convenient source of NO_3_, especially compared to whole-food sources, and is now considered a key research area in this field.

Whilst awareness of NO_3_ supplementation is now increasing, it is not clear if its effects are age-dependent. Evidence suggests that vascular cells experience reduced endothelium-dependent dilation following inducement with age [3, 4]. Data also indicates that younger adults might respond differently to dietary intervention than older individuals [15]. Despite this, data suggests that aged endothelium can maintain responsivity to dietary and lifestyle factors [16]. This is important, as non-pharmacological treatments could be a welcome alternative for some older patients, if tolerable and efficacious.

Therefore, the aim of this research is to investigate the effects of dietary NO_3_ ingestion, in the form of beetroot juice supplementation, on cardiovascular and microvascular endothelial function in younger and older adults.

## Materials and methods

### Study design

A randomised, placebo-controlled, double-blind, crossover, pilot study was undertaken to evaluate the effects of acute beetroot juice supplementation in younger and older adults. Forty-eight individuals volunteered to participate. Due to budget and time constraints, sample size was based on practicality, with a view to recruit ~30 participants. Inclusion criteria were established a priori to include healthy, normotensive participants of 18–35 years or ≥55 years of age with SBP between 115 and 140 mmHg and DBP between 75 and 90 mmHg. Exclusion criteria of anti-hypertensive medication treatment, heart disease, diabetes or other diseases that impact cardiovascular function, the consumption of antacids or proton pump inhibitors, smoking, weight loss of more than three kilograms in the month before recruitment, and or incomplete denture, were executed. Participants were screened prior to inclusion, provided written informed consent, and were randomised electronically using nQuery (GraphPad Software, San Diego, CA). The study was approved by the ethics committee of Sheffield Hallam University and registered at ClinicalTrials.gov (Identifier: NCT04276766). All work was carried out in accordance with the Declaration of Helsinki. Figure one provides a Consolidated Standards of Reporting Trials (CONSORT) overview of the study design and procedures.

### Anthropometric measurements

Stature (cm), Body Mass (kg), Body Mass Index (kg m^2^), Waist Circumference (cm) and Body Fat Mass (% fat mass) were collected at baseline (Visit 1). Body composition was assessed via bioelectrical impedance analysis (Tanita MC-780MA P, TANITA, Japan).

### Intervention and placebo

Both groups received 70 ml of concentrated beetroot juice or a NO_3_-absent placebo (James White Drinks Company Suffolk, UK) during visits 2 and 3 in a counterbalanced order. Previous data indicates that a beetroot juice providing 5.5 mmol NO_3_ was sufficient to elicit a reduction in BP using methods analogous to this study [13]. The treatment therefore consisted of 70 ml of beetroot juice containing ~6.4 mmol of NO_3_. The placebo matched the organoleptic qualities of the treatment. Participants and researchers were blinded to the administration of the supplementation.

### Procedures

Urine samples, blood pressure and microcirculatory endothelial function was assessed at the start of each visit prior to the ingestion of the drinks. Earlier data indicates that beetroot juice supplementation can reduce blood pressure within 2 h following ingestion, reflecting the time to produce nitrite from NO_3_ via the entero-salivary circulation [13]. Consequently, we measured blood pressure and heart rate every 20 min for 3 h following ingestion. Blood pressure assessments were then followed by urine sample collection and microcirculatory endothelial assessment. A low NO_3_ meal was provided to participants for consumption later that day (pasta [75 g uncooked weight] with tomato sauce). Urine samples were collected the following morning at the participants’ residence with the samples transported to the laboratory on the day of collection. The third visit consisted of a crossover assessment in the same order. Female participants were assessed during the same phase of their menstrual cycle to avoid hormonal fluctuation-induced alterations in vascular tone.

### Blood pressure

Blood pressure was assessed in a seated position with the participant sat resting for 15 min prior to measurement. Blood pressure readings were taken three times using an automated monitoring device (Dinamap Dash 2500, Ge Healthcare, USA) and mean score was calculated and used within our analyses: $\bar x = \frac{{\left( {\sum x} \right)}}{n}$.

### Microcirculatory endothelial function

Laser Doppler Flowmetry (LDF) evaluated microcirculatory endothelial function pre and post supplementation. Measurements were taken in the upper left forearm as previously described [17]. Briefly, a 7-point integrating LDF probe (probe 413, 123 Perimed AB) and local heating disc were used (Periflux system 5000, Perimed 122 AB, Jarfalla, Sweden). The probe and the heating disc were attached to the forearm of the participant. Baseline measurement was taken with the heating disc set to 32 °C for 5 min. Temperature then increased by 1 °C every 10 s until the disc reached 42 °C, which was then maintained for 25 min to obtain an initial peak measurement. Finally, the temperature was rapidly increased to 44 °C for 10 min to obtain maximal vasodilatation. Data was divided by the mean arterial pressure and expressed as cutaneous vascular conductance (CVC) in APU/mmHg. CVC values were normalised to the percentage of maximal cutaneous vascular conductance and calculated as $\% {\it{CVCmax}} = \left( {\frac{{CVC}}{{{\it{maximum}}\,{\it{CVC}}}}} \right) \times 100$.

### Urinary nitric oxide content

Urinary NO_3_ was measured via midstream urine samples collected on three occasions: (1) pre supplementation; (2) 3 h post supplementation; (3) 24-h post supplementation. Collected samples were stored at −80 °C. NO content was measured by the Griess reaction [18] using a NO_3_ Colorimetric Assay Kit (Sigma-Aldrich, St.Lous, USA).

### Habitual diet and exercise

Participants were instructed to avoid physical activity, maintain hydration and refrain from chewing gum or mouthwash 3 days prior to each visit, to maintain the oral microbiome and minimise disruption to the nitrate-nitrate pathway [18–20]. Participants were instructed to consume a low- NO_3_ diet (to achieve ~25–50 mg NO_3_ per day^−1^) and provided with low-NO_3_ water (e.g., Buxton Water <0.1 mg NO_3_. L) to consume the day prior to each visit. Participants were provided with meal ingredients (chicken fillets, pasta, eggs, and low-NO_3_ water) as well as recommendations to consume low-nitrate foods (such as fresh chicken and meat, eggs, tomatoes, peppers, green beans, potatoes, asparagus, mushrooms, and peas), avoid foods high in nitrate (green leafy vegetables, beetroot, radishes, and cured meats), and suggested meals to facilitate achievement of values ~25–50 mg NO_3_ per day^−1^ in the 24 h prior to assessment (such as scrambled eggs, fresh meat, pasta and potato dishes). Participants abstained from eating prior to each visit. Visits 2 and 3 were separated by a 7-day washout period.

### Statistical analyses

Data was assessed for assumptions prior to inferential analysis. Residual assessment revealed the presence of three outliers in the younger group’s data, who were removed from the dataset to satisfy the assumptions of ANOVA. Baseline characteristics were compared between groups using an independent-samples *t*-test (Young vs. Old). Following this, a three-way, mixed-design ANOVA with two between-groups (Treatment [NO_3_ vs. Placebo] and Age [Young and Old]) and one within-groups factor (time) was undertaken for Blood Pressure, Heart Rate and LDF data. Urinary NO_3_ data were analysed via a Mann–Whitney *U* test due to violations of normality. Confidence Intervals of 75–95% were calculated along with the Minimally Important Change (MIC) and can be found in the supplementary information (Supplementary Tables 1a–3). Data are expressed as mean ± SD unless otherwise stated. Inferential analysis was carried out using SPSS 26.

## Results

### Participants

**Fig. 1:**
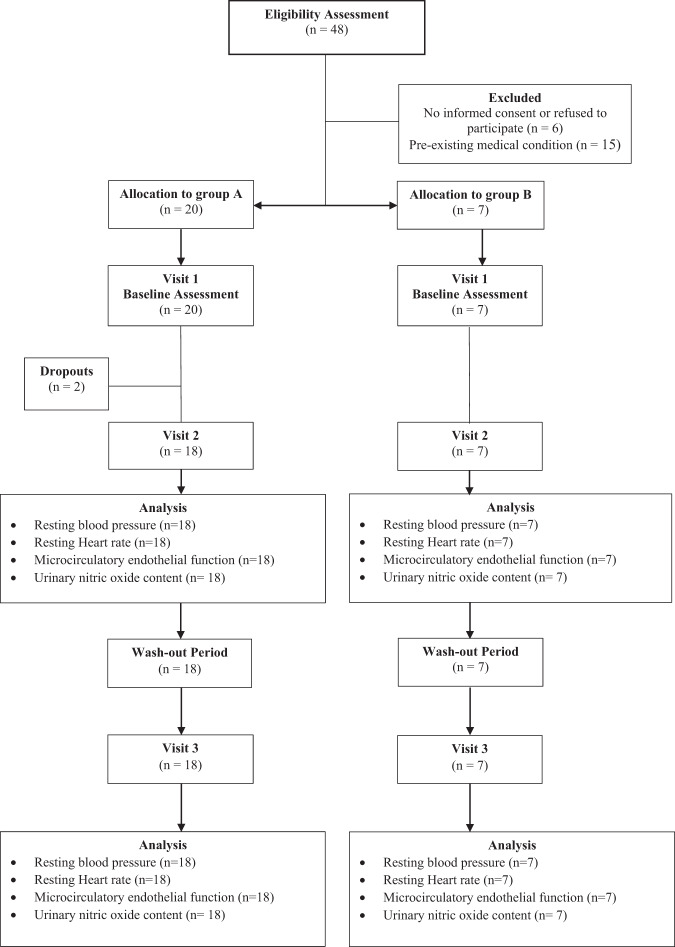
Study design and procedures. Consolidated standards of reporting trials (CONSORT) flow diagram showing participant flow throughout the study.

Twenty-seven participants met our criteria, were randomised to the intervention, however two withdrew following Visit 1 (Fig. 1). Twenty-five participants completed the study, no adverse effects were reported, and the intervention, placebo and assessments were well tolerated. Participants in group A/Young had a lower BMI (22.7 ± 2.8 vs. 26.7 ± 5.1 kg m^2^, *P* = 0.019), WC (78.9 ± 8.1 vs. 96.4 ± 15.8 cm, *P* = 0.001), resting heart rate (67.8 ± 14.9 vs 96.0 ± 42.5 bpm, *P* = 0.020) and DBP than participants in group B/Old (66.8 ± 9.9 vs. 81.3 ± 10.9 mmHg, *P* = 0.004) during the study. Participants’ baseline characteristic data can be found in Table 1.

**Table 1:** Baseline anthropometric and blood pressure data: pre-treatment comparison between Young (*n* = 17) vs. Old (*n* = 7) participants prior to administration of NO_3_ supplementation.

	YOUNG (*n* = 17)		OLD (*n* = 7)	
	Mean	SD	Mean	SD
Age (y)*	24.2	3.5	68.7	10.3
*P* =	0.000			
Stature (cm)	168.3	7.9	168.3	10.2
*P* =	0.997			
Body mass (kg)	62.7	12	76.7	20.7
*P* =	0.193			
BMI (kg m^2^)**	22.7	2.8	26.7	5.1
*P* =	0.019			
WC (cm)**	78.8	8.1	96.4	15.8
*P* =	0.001			
Body fat (%)	15.9	5.9	22.9	11.4
*P* =	0.051			
SBP (mmHg)	106.8	20	115.1	28.4
*P* =	0.415			
DBP (mmHg)**	66.8	9.9	81.3	10.9
*P* =	0.004			
RHR (bpm)**	67.8	14.9	96	42.5
*P* =	0.020			

Independent-samples *t*-test reveals between-groups differences at the 0.001 (*) and 0.005 level (**) for Age*, BMI**, WC**, DBP** and RHR**.

*WC* waist circumference, *SBP* systolic blood pressure, *DBP* diastolic blood pressure, *RHR* resting heart rate.

### Blood pressure and heart rate

**Fig. 2:**
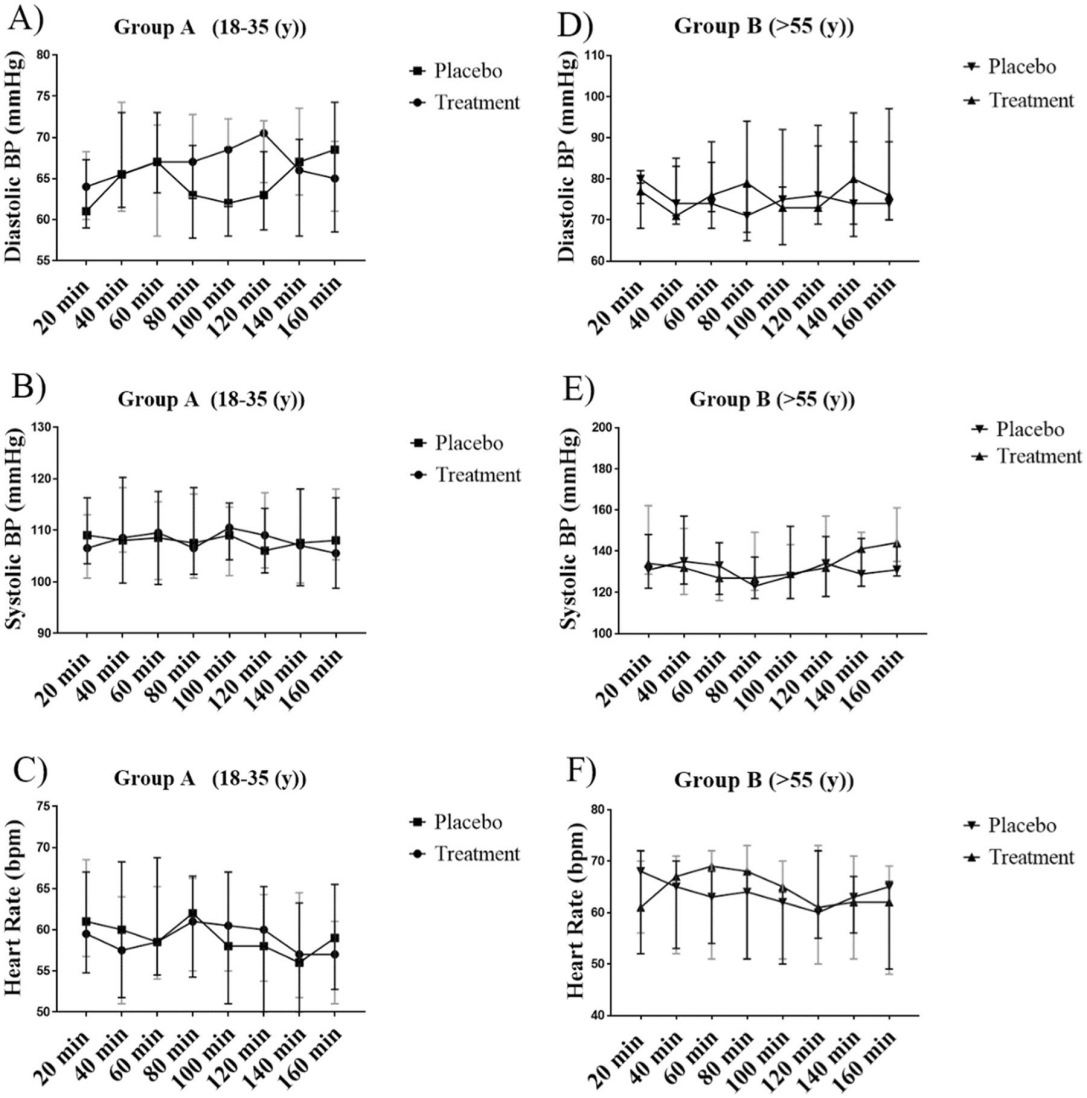
Blood pressure and heart rate following Treatment vs. Placebo. Three-way mixed ANOVA, with two between groups (Treatment vs. Placebo and Age [Young/Group A vs. Old/Group B]) and one within group factor (time), revealed no effects of NO_3_ supplementation (*P* > 0.05) for Group A (Young: *n* = 17) nor Group B (Old: *n* = 7).

Beetroot juice had no effect on SBP, DBP or heart rate in either group [(*P* ≥ 0.05) Fig. 2] and no interactions between variables were noted. Differences in mean blood pressure and heart rate scores between conditions can be found in Table 2.

**Table 2:** Difference between placebo vs. nitrate blood pressure, heart rate and skin blood flow responses in Young (*n* = 17) vs. Old (*n* = 7) participants.

	YOUNG (*n* = 17)		OLD (*n* = 7)	
	Mean	SD	Mean	SD
Δ SBP	−0.74	1.3	30.4	8
Δ DBP	1.4	1	16	4
Δ HR	0.4	1.1	−1.2	4.6
CVC Baseline*				
Δ Nitrate	2.6	6.9	6.1	5.5
Δ Placebo	3.9	3.8	−1	6.5
CVC Initial Peak				
Δ Nitrate	16.2	43.9	9.7	25.8
Δ Placebo	17.5	28.4	−1	40.9
CVC Plateau				
Δ Nitrate	−1.9	11.4	−2.4	7.4
Δ Placebo	−5.2	10.9	−1.5	6.6

Statistically significant interaction at the *P* ≤ 0.05 level (*) observed between age and treatment for CVC at baseline (*P* = 0.037) only.

### Microvascular endothelial function

There was a significant interaction between age and treatment at baseline CVC (*F* [1, 37] = 4.7, *P* = 0.037). Specifically, older participants exhibited a reduction in baseline CVC between treatments (Placebo = 12.2 ± 4.4, NO_3_ = 8.7 ± 7.0). No effects were observed at the initial peak (*P* ≥ 0.05) nor the plateau stages (*P* ≥ 0.05) in either group, however. Differences in pre-to-post CVC data between conditions can be found in Table 2.

### Urinary nitric oxide

Beetroot juice significantly increased urinary NO_3_ at 3 h [489.7 ± 128 µM (*P* = 0.02)] and after 24 h [332.9 ± 102.6 µM (*P* = 0.02)] following consumption in younger participants. Beetroot juice increased urinary NO_3_ at 3 h [386 ± 152.2 µM (*P* = 0.02)] but not after 24 h (*P* ≥ 0.05) in older participants. Urinary NO_3_ levels did not differ between younger and older participants at 3 h (*P* ≥ 0.05) nor 24 h (*P* ≥ 0.05).

## Discussion

The results of this pilot study revealed that the treatment, placebo, and methods were feasible and well tolerated, but that a single dose of beetroot juice had no effect on blood pressure, heart rate or microvascular endothelial function in younger and older individuals. This was surprising, considering that NO_3_ supplementation has previously improved such parameters [8–14, 21–23]. We hypothesise that the lack of effects observed might be explained by inadequate NO_3_ dosing, indicating that methodological limitations rather than inadequate treatment efficacy explain these results. Coles et al. reported that beetroot juice supplementation led to a 4.6 mmHg reduction in SBP 3 h after consuming 15 mmol of NO_3_ (which later increased to 6.2 mmHg 6 h post ingestion) [21]. This contrasts markedly with the 6.4 mmol that we provided and the 5.5 mmol provided by Kapil et al. [12]. In this latter study, 5.5 mmol led to reduced SBP using methods that mirror this research. Whilst the lack of effects we observed were disappointing, we posit that a dosing threshold might exist for reliable changes in blood pressure to be detected.

Wylie et al. [12] observed that 8.4 mmol and 16.8 mmol of NO_3_ elicited reduced blood pressure but that a 4.2 mmol dose prompted no obvious benefit. We observed no change employing a 6.4 mmol dose.Therefore, it is possible that a minimum effective dose for NO_3_ is above 6.4 mmol but equal to or below the 8.4 mmol employed in Wylie et al.’s work, contrasting with the 5.5 mmol does of Kapil’s work. Further work is perhaps needed to elucidate dosing thresholds such that future studies can be assured of achieving an appropriate level of efficacy.

We monitored blood pressure and microvascular responses for 3 h following ingestion of the juice. It is also possible that this window might have been insufficient for the full effects of the ingested NO_3_ to be realised. Previous research has shown that NO_3_ is time sensitive and that changes in blood pressure and microvascular function are maximised 6 h post ingestion. However, previous work has also highlighted improvements in blood pressure and cardiovascular outcomes manifest within 2–3 h following doses within a 5.5–16.8 mmol range [12, 13, 21]. Nevertheless, most studies observing acute effects have done so, following larger amounts. Therefore, whilst the 3-h window might have been sufficient to observe changes in the parameters we measured, the 6.4 mmol dose might be too small to elicit them observably within this timeframe. Further work is needed to explore timeframes beyond 3 h at this lower dose, particularly in comparison to other doses and timeframes to determine if dose x time interactions do occur, and if more time is needed for smaller doses to realise their effects. We expect that such findings would be of interest to researchers in this field and that future investigations employ NO_3_ doses >6.4 mmol if measuring responses within a 3-h window.

Ageing leads to stiffening of the large arteries, resulting in an increase in SBP, a decrease in DBP and diminished responsiveness to NO, which could be an important consideration, if using NO-based treatments for cardiovascular conditions [2, 3]. Contrary to our expectations, we observed no differences between age groups. Previous data from our lab indicated that older and younger adults differ in responses to dietary modifications that ameliorate microvascular health [15]. In this earlier work, we observed greater responses in younger participants whilst investigating the effects of a novel diet [15]. Interestingly, Jones et al. observed no change in endothelial function in older individuals following 4 weeks of beetroot juice supplemenation [23]. However, Wong et al. observed no change in microvascular function following a 3-day period in young volunteers however [24]. This evidence suggests a lack of age-related effects. Wong et al. discuss that sensory nerves initiate vasodilation and that supplemental NO_3_ has no effect on sensory-nerve-mediated dilation, leading to poor responses if using techniques that measure this response [24]. Our findings might echo Wong’s suggestions and indicate that NO_3_ supplementation has no effect on cutaneous reactive hyperaemia in younger and older adults via measurements of cutaneous reactive hyperaemia. However, research employing different methods offer similar findings too, suggesting that these effects are not an instrumentation issue—Gilchrist et al. observed no effect on macro and microvascular function using laser Doppler imaging in older participants in their study [25]. Collectively, evidence could indicate that beetroot juice does not affect microvascular function using techniques found in the literature.

A strength of this study were the measures we took to control NO_3_ intake prior to assessment. This was necessary to minimise the effects of habitual consumption of NO_3_-rich foods, which could lead to an additive effect when combined with the beetroot juice, or insufficient disparity in NO_3_ consumption between groups or conditions. Despite this, we observed a reduction in baseline CVC in older participants between treatments. Indeed, we took measures to promote the consumption of a NO_3_-depleted diet prior to assessment, including the provision of foodstuffs (poultry, eggs, pasta), meal suggestions (fresh meat, pasta, potato, and egg-based dishes) and a list of foods to avoid (such as leafy green vegetables and other NO_3-_rich foods such as beetroot). Despite this, it is possible that some older participants adjusted their habitual diet throughout the study period to the extent that this affected baseline CVC between conditions. It has been remarked elsewhere that few NO_3_ studies have administered NO_3_-depleted diets during experimentation [25]. A lack of measures taken to control and monitor the diet could therefore be obscuring literature understanding of NO_3_ supplementation. We have hypothesised previously that nutritional elements interact within a whole-food diet to augment the hypotensive and microvascular responses of an intervention [26]. Indeed, Gilchrist note that participants in the DASH study experienced greater hypotensive effects when total and saturated fat intakes were reduced alongside an increase in fruits and vegetables [25, 27]. We observed similar findings when comparing a Vegan diet to a Mediterranean diet and hypothesised that the consumption of olive oil might augment the effects of the Mediterranean diet, which is abundant in NO_3_-rich foods [26]. This is important, as it is conceivable that the inconsistencies in response to beetroot juice supplementation observed in the literature are due to dietary components interacting, and that if participants are consuming a diet rich in NO_3,_ that the effects of supplemental NO_3_ are augmented. Indeed, isolating the effects of an intervention in free living conditions is challenging, and it is likely that there are elements of the diet that interact in such ways that are not known nor observable to date, and that these effects could be confounding data. Research teams should therefore be mindful of the effects of habitual diets when administering interventions in free living conditions, and perhaps seek ways to control and assesses dietary factors to partition out their effects in subsequent studies.

We noted that urinary NO_3_ levels remained elevated 24 h post ingestion in the young participants only despite both groups experiencing elevated values at 3 h. This finding was interesting and perhaps indicates an age or lifestyle-related factor leading to greater rates of excretion in older individuals. Regular consumption of NO_3_-rich foods can increase its rate of excretion [28], which is consistent with our supposition that older participants might have increased their habitual intake between visits and explaining the disparity in 24-h concentrations between groups. This reinforces our suggestion for future research, to carefully monitor NO_3_ consumption during treatment.

A limitation of this study was the small sample size, as due to technical and budget restrictions we were not able to recruit equal group sizes. A sample size calculation was also not performed for this study, and therefore we are unable to confirm whether the findings are adequately powered. Nevertheless, it is important to note that testing hypotheses within pilot studies, should be viewed as preliminary only, owing to statistical power not being a prerequisite nor null hypothesis testing a requirement [28]. Indeed, sample size in pilot studies are often based on pragmatics [28], however findings can provide an initial assessment of clinical benefit [29]. It has also been recommended that pilot studies provide descriptive data and confidence interval estimates such that decisions can be made with regards to further investigation in the area [9, 29]. These can be found in Supplementary Tables 1a–3. We therefore recommend that our inferential results are interpreted cautiously. Issues of dosing, timing, and methods to assess microvascular function and habitual lifestyle factors have all been highlighted as being important considerations for future studies within this line of research. These factors should be considered in future investigations that wish to compare responses in younger and older adults.

## Conclusions

Preliminary findings of this pilot study indicate that the treatment and assessments methods were feasible and well tolerated, and that a single dose of dietary NO_3_ had no effect on cardiovascular function in younger and older adults. A range of factors were highlighted that could explain the lack of treatment effect observed, including dosing, timing of follow-up blood pressure assessments and habitual diet, which need to be considered in future investigations if treatment efficacy is to be guaranteed. We suggest that future work should be undertaken to elucidate the minimum effective dose for NO_3_ supplementation and that more work is needed to explore dose x time interactions. Such findings are perhaps important if further work is to continue in this area.

## Supplementary information

Supplemental Material

## Data Availability

The datasets generated during and/or analysed during the current study are available from the corresponding author on reasonable request.

## References

[1] Zhao Y, Vanhoutte PM, Leung SW (2015). Vascular nitric oxide: Beyond eNOS. J Pharm Sci..

[2] Yazdanyar A, Newman AB (2009). The burden of cardiovascular disease in the elderly: morbidity, mortality, and costs. Clin Geriatr Med..

[3] James MA, Tullett J, Hemsley AG, Shore AC (2006). Effects of aging and hypertension on the microcirculation. Hypertension..

[4] Rossman MJ, Kaplon RE, Hill SD, McNamara MN, Santos-Parker JR, Pierce GL (2017). Endothelial cell senescence with aging in healthy humans: prevention by habitual exercise and relation to vascular endothelial function. Am J Physiol Heart Circ Physiol..

[5] Pickering TG (2005). Why don’t we use nitrates to treat older hypertensive patients?. J Clin Hypertens.

[6] Gladwin MT, Crawford JH, Patel RP (2004). The biochemistry of nitric oxide, nitrite, and hemoglobin: role in blood flow regulation. Free Radic Biol Med..

[7] Bailey JC, Feelisch M, Horowitz JD, Frenneaux MP, Madhani M (2014). Pharmacology and therapeutic role of inorganic nitrite and nitrate in vasodilatation. Pharm Ther..

[8] Lauer T, Preik M, Rassaf T, Strauer BE, Deussen A, Feelisch M (2001). Plasma nitrite rather than nitrate reflects regional endothelial nitric oxide synthase activity but lacks intrinsic vasodilator action. Proc Natl Acad Sci USA.

[9] Webb AJ, Patel N, Loukogeorgakis S, Okorie M, Aboud Z, Misra S (2008). Acute blood pressure lowering, vasoprotective, and antiplatelet properties of dietary nitrate via bioconversion to nitrite. Hypertension..

[10] Larsen FJ, Ekblom B, Sahlin K, Lundberg JO, Weitzberg E (2006). Effects of dietary nitrate on blood pressure in healthy volunteers. N. Engl J Med..

[11] Wylie LJ, Kelly J, Bailey SJ, Blackwell JR, Skiba PF, Winyard PG (2013). Beetroot juice and exercise: pharmacodynamic and dose-response relationships. J Appl Physiol..

[12] Kapil V, Milsom AB, Okorie M, Maleki-Toyserkani S, Akram F, Rehman F (2010). Inorganic nitrate supplementation lowers blood pressure in humans: role for nitrite-derived NO. Hypertension.

[13] Kim JK, Moore DJ, Maurer DG, Kim-Shapiro DB, Basu S, Flanagan MP (2015). Acute dietary nitrate supplementation does not augment submaximal forearm exercise hyperemia in healthy young men. Appl Physiol Nutr Metab..

[14] Rogerson D, McNeill S, Könönen H, Klonizakis M (2018). Encouraging effects of a short-term, adapted Nordic diet intervention on skin microvascular function and skin oxygen tension in younger and older adults. Nutrition..

[15] Klonizakis M, Alkhatib A, Middleton G (2014). Long-term effects of an exercise and Mediterranean diet intervention in the vascular function of an older, healthy population. Microvasc Res..

[16] Bryan NS, Grisham MB (2007). Methods to detect nitric oxide and its metabolites in biological samples. Free Radic Biol Med..

[17] Wasilewski R, Ubara EO, Klonizakis M. Assessing the effects of a short-term green tea intervention in skin microvascular function and oxygen tension in older and younger adults. Microvasc Res. 2016 Sep;107:65-71. 10.1038/s41598-020-61912-4. 10.1016/j.mvr.2016.05.001 27165772

[18] Bescos R, Ashworth A, Cutler C, Brookes ZL, Belfield L, Rodiles A. et al. Effects of Chlorhexidine mouthwash on the oral microbiome. Sci Rep. 2020;10:5254. 10.1038/s41598-020-61912-4. 10.1038/s41598-020-61912-4 PMC7093448 32210245

[19] van Maanen JM, van Geel AA, Kleinjans JC. Modulation of nitrate-nitrite conversion in the oral cavity. Cancer Detect Prev. 1996;20:590–6. 8939344

[20] Sim J. Should treatment effects be estimated in pilot and feasibility studies?. Pilot Feasibility Stud. 2019;5:107. 10.1186/s40814-019-0493-7. 10.1186/s40814-019-0493-7 PMC6712606 31485336

[21] Siervo M, Lara J, Ogbonmwan I, Mathers JC (2013). Inorganic nitrate and beetroot juice supplementation reduces blood pressure in adults: a systematic review and meta-analysis. J Nutr..

[22] Jones T, Dunn EL, Macdonald JH, Kubis HP, McMahon N, Sandoo A (2019). The Effects of Beetroot Juice on Blood Pressure, Microvascular Function and Large-Vessel Endothelial Function: A Randomized, Double-Blind, Placebo-Controlled Pilot Study in Healthy Older Adults. Nutrients..

[23] Wong BJ, Keen JT, Levitt EL (2018). Cutaneous reactive hyperaemia is unaltered by dietary nitrate supplementation in healthy humans. Clin Physiol Funct Imaging.

[24] Gilchrist M, Winyard PG, Aizawa K, Anning C, Shore A, Benjamin N (2013). Effect of dietary nitrate on blood pressure, endothelial function, and insulin sensitivity in type 2 diabetes. Free Radic Biol Med..

[25] Rogerson D, Maçãs D, Milner M, Liu Y, Klonizakis M (2018). Contrasting Effects of Short-Term Mediterranean and Vegan Diets on Microvascular Function and Cholesterol in Younger Adults: A Comparative Pilot Study. Nutrients..

[26] Svetkey LP, Simons-Morton D, Vollmer WM, Appel LJ, Conlin PR, Ryan DH (1999). Effects of dietary patterns on blood pressure: subgroup analysis of the Dietary Approaches to Stop Hypertension (DASH) randomized clinical trial. Arch Intern Med..

[27] Lancaster GA, Dodd S, Williamson PR (2004). Design and analysis of pilot studies: recommendations for good practice. J Eval Clin Pract..

[28] Hill MJ, Elliott P, Joossens JV, Packer PJ, Kesteloot H, Nichols R (1996). Twenty-four hour urinary nitrate excretion in 48 populations from 30 countries: an ECP-INTERSALT collaborative study. Int J Epidemiol..

[29] Lee EC, Whitehead AL, Jacques RM, Julious SA (2014). The statistical interpretation of pilot trials: should significance thresholds be reconsidered?. BMC Med Res Methodol..

