# Supplementary material for: No effect of nitrate-rich beetroot juice on microvascular function and blood pressure in younger and older individuals: a randomised, placebo-controlled double-blind pilot study
Source: Eur J Clin Nutr. 2022 Mar 29;76(10):1380–6. doi: 10.1038/s41430-022-01115-4 (PMC9550618; doi:10.1038/s41430-022-01115-4)
Supplement: Supplementary file 1 — Supplemental Material [file 41430_2022_1115_MOESM1_ESM.docx]

**SUPPLEMENTARY MATERIAL**

|  | Age (years) | | Stature (cm) | | Body Mass (kg) | | BMI | | WC (cm) | | Body Fat (%) | |
| --- | --- | --- | --- | --- | --- | --- | --- | --- | --- | --- | --- | --- |
|  | **Young**  **(n = 17)** | **Old**  **(n = 7)** | **Young**  **(n = 17)** | **Old**  **(n = 7)** | **Young**  **(n = 17)** | **Old**  **(n = 7)** | **Young**  **(n = 17)** | **Old**  **(n = 7)** | **Young**  **(n = 17)** | **Old**  **(n = 7)** | **Young**  **(n = 17)** | **Old**  **(n = 7)** |
| 95% CI | 22.4 25.9 | 59.2 78.2 | 164.4 172.2 | 158.9 177.7 | 58.9 70.8 | 57.6 95.8 | 21.3 24.1 | 22.0 31.4 | 74.8 82.9 | 81.8 111.1 | 13.0 18.8 | 12.4 33.5 |
| 90% CI | 22.7 25.6 | 61.2 76.3 | 165.1 171.6 | 160.8 175.8 | 59.9 69.8 | 61.5 91.9 | 21.6 23.4 | 23.0 30.4 | 75.5 82.2 | 84.8 108.1 | 13.5 18.3 | 14.6 31.3 |
| 85% CI | 22.9 25.4 | 62.3 75.13 | 165.5 171.1 | 161.9 174.6 | 60.6 69.1 | 63.4 89.6 | 21.7 23.7 | 23.5 29.9 | 76.0 81.8 | 86.5 106.3 | 13.8 18.0 | 15.8 30.0 |
| 80% CI | 23.1 25.3 | 63.1 74.3 | 165.8 170.8 | 162.7 173.8 | 61.1 68.6 | 65.4 87.9 | 21.9 23.6 | 24.0 29.5 | 76.3 81.4 | 87.8 105.1 | 14.1 17.7 | 16.7 29.1 |
| 75% CI | 23.2 25.2 | 63.8 73.7 | 166.1 170.5 | 163.4 173.2 | 61.5 68.2 | 66.7 86.6 | 22.0 23.5 | 24.3 29.2 | 76.6 81.2 | 88.8 104.1 | 14.3 17.5 | 17.4 28.4 |

**WC =** Waist circumference.

**Supplemental Table 1a.** Baseline 75% - 95% confidence intervals for anthropometric data in Young (n = 17) vs. Old (n = 7) participants prior to administration of NO_3_ supplementation. Confidence intervals calculated to reflect descriptive statistic emphasis recommendations for pilot studies^20^, and supplement means and SD of the main manuscript.

|  | SBP | | DBP * | | RHR* | |
| --- | --- | --- | --- | --- | --- | --- |
|  | **Young**  **(n = 17)** | **Old**  **(n = 7)** | **Young**  **(n = 17)** | **Old**  **(n = 7)** | **Young**  **(n = 17)** | **Old**  **(n = 7)** |
| 95% CI | 96.9 116.8 | 88.9 141.4 | 61.8 71.7 | 71.2 91.4 | 60.4 75.3 | 56.7 135.3 |
| 90% CI | 98.6 115.0 | 94.3 136.0 | 62.7 70.9 | 73.3 89.3 | 61.7 74.0 | 64.8 127.2 |
| 85% CI | 99.7 113.9 | 97.4 132.9 | 63.2 70.3 | 74.5 88.1 | 62.5 73.2 | 69.5 122.5 |
| 80% CI | 23.1 25.3 | 63.1 74.3 | 165.8 170.8 | 162.7 173.8 | 63.1 72.5 | 63.0 72.5 |
| 75% CI | 23.2 25.2 | 63.8 73.7 | 166.1 170.5 | 163.4 173.2 | 61.5 68.2 | 66.7 86.6 |

**SBP =** Systolic blood pressure, **DBP =** Diastolic blood pressure, **RHR** = Resting heart rate.

**Supplemental Table 1b.** Baseline 75% - 95% confidence intervals for blood pressure in Young (n = 17) vs. Old (n = 7) participants prior to administration of NO_3_ supplementation.

Confidence intervals calculated to reflect descriptive statistic emphasis recommendations for pilot studies^20^, and supplement means and SD of the main manuscript.

| Young (n = 17) | | | | | |
| --- | --- | --- | --- | --- | --- |
|  | | **Δ SBP** | **Δ DBP** | **Δ HR** |  |
| MIC | | 2.8 | 2.1 | 2.4 |  |
| 95% CI | Lower  Upper | -3.5  2.0 | -0.7  3.5 | -2.0  2.8 |  |
| 90% CI | Lower  Upper | -3.0  2.0 | -0.3  3.2 | -1.6  2.4 |  |
| 85% CI | Lower  Upper | -2.7  1.3 | -0.1  2.9 | -1.3  2.1 |  |
| 80% CI | Lower  Upper | -2.5  1.0 | 0.1  2.8 | -1.1  1.9 |  |
| 75% CI | Lower  Upper | -2.3  0.8 | 0.2  2.6 | -0.9  1.8 |  |
| Old (n = 7) | | | | | |
|  | | **Δ SBP** | **Δ DBP** | **Δ HR** |  |
| MIC | | 10.6 | 5.3 | 6.1 |  |
| 95% CI | Lower  Upper | 10.7  50.0 | 6.3  25.7 | -12.4  10.1 |  |
| 90% CI | Lower  Upper | 14.8  46.0 | 8.2  23.7 | -10.1  7.8 |  |
| 85% CI | Lower  Upper | 17.1  43.6 | 9.4  22.5 | -8.8  6.4 |  |
| 80% CI | Lower  Upper | 18.8  41.9 | 10.3  21.7 | -7.8  5.5 |  |
| 75% CI | Lower  Upper | 20.2  40.6 | 10.9  21.1 | -7.0  4.7 |  |

**Supplemental Table 2.** 75% - 95% confidence intervals and minimally important change for difference between placebo vs. nitrate blood pressure and heart rate in Young (n = 17) vs. Old (n = 7) participants.

Minimally Important Change (MIC) calculated via SEM.

Confidence intervals and MIC data for Δ SBP and DBP could indicate a clinically important treatment difference warranting further investigation via a further confirmatory trial^20^, supplementing the inferential analyses of the main manuscript.

| Young (n = 17) | | | | | | | |
| --- | --- | --- | --- | --- | --- | --- | --- |
|  | | **Baseline** | | **Initial Peak** | | **Plateau** | |
|  | | **Δ Nitrate** | **Δ Placebo** | **Δ**  **Nitrate** | **Δ**  **Placebo** | **Δ**  **Nitrate** | **Δ Placebo** |
| MIC | | 1.8 | 1.1 | 11.3 | 7.9 | 2.9 | 3.0 |
| 95% CI | Lower  Upper | -1.2  6.4 | 1.6  6.2 | -8.1  40.5 | 0.3  34.7 | -8.2  4.5 | -11.7  1.4 |
| 90% CI | Lower  Upper | -0.5  5.7 | 2.0  5.8 | -3.8  36.2 | 3.5  31.5 | -7.1  3.3 | -10.5  0.2 |
| 85% CI | Lower  Upper | -0.1  5.3 | 2.3  5.6 | -1.1  33.5 | 5.4  29.6 | -6.4  2.6 | -9.8  -0.5 |
| 80% CI | Lower  Upper | 0.2  5.0 | 2.5  5.4 | 1.0  31.5 | 6.8  28.2 | -5.8  2.1 | -9.3  -1.1 |
| 75% CI | Lower  Upper | 0.5  4.7 | 2.7  5.2 | 2.6  29.8 | 8.0  27.0 | -5.4  1.7 | -8.8  -1.5 |
| Old (n = 7) | | | | | | | |
|  | | **Baseline** | | **Initial Peak** | | **Plateau** | |
|  | | **Δ Nitrate** | **Δ Placebo** | **Δ**  **Nitrate** | **Δ**  **Placebo** | **Δ**  **Nitrate** | **Δ Placebo** |
| MIC | | 2.2 | 2.5 | 10.5 | 15.5 | 3.0 | 2.5 |
| 95% CI | Lower  Upper | 0.3  11.8 | -7.0  5.0 | -17.4  36.8 | -38.9  36.8 | -10.2  5.4 | -7.6  4.6 |
| 90% CI | Lower  Upper | 1.6  10.6 | -5.8  3.8 | -11.5  31.0 | -31.1  29.0 | -8.5  3.7 | -6.3  3.4 |
| 85% CI | Lower  Upper | 2.3  9.9 | -5.1  3.0 | -8.2  27.6 | -26.6  24.5 | -7.6  2.7 | -5.6  2.6 |
| 80% CI | Lower  Upper | 2.8  9.4 | -4.6  2.5 | -5.9  25.3 | -23.3  21.2 | -6.9  2.1 | -5.1  2.1 |
| 75% CI | Lower  Upper | 3.2  9.0 | -4.2  2.1 | -4.0  23.4 | -20.7  18.7 | -6.3  1.5 | -4.7  1.7 |

**Supplementary Table 3.** 75% - 95% confidence intervals and minimally important change for skin blood flow in Young (n = 17) vs. Old (n = 7) participants.

Confidence intervals and MIC for Δ Nitrate and Δ Placebo could indicate a clinically important treatment difference for Baseline CVC in Old participants^20^ and supplement the inferential analyses of the main manuscript.
